# Oil and Water

**DOI:** 10.1289/ehp.1002953

**Published:** 2010-10

**Authors:** Elizabeth T.H. Fontham, Edward Trapido

**Affiliations:** Louisiana State University Health Sciences Center, School of Public Health, New Orleans, Louisiana, E-mail: efonth@lsuhsc.edu

Disasters and their environmental and health consequences know no bounds. Although no geographic area or population is exempt from natural or manmade disasters, Louisiana and its neighboring coastal states on the Gulf of Mexico have had more than their fair share over the past 5 years. The latest environmental disaster is the BP *Deepwater Horizon* oil spill, which began on 20 April 2010, < 5 years after Hurricanes Katrina and Rita wreaked havoc in the same area. The effects of hurricanes, which are annual events, are relatively well understood, but the environmental and human health effects of oil spills are much less so.

The amount of oil released from the spill site peaked at about 62,000 barrels/day (Robertson and Krauss 2010) until the well was permanently capped on 15 July 2010. The U.S. Geological Survey (USGS 2010) estimated that as of 1 August 2010, 4.4–5.4 million barrels of oil were released. In addition, approximately 1 billion ft^3^ of natural gas has been flared, well over 265,000 barrels of oil have been burned, and > 1.8 million gallons of dispersant (primarily Corexit 9500 but also Corexit 9527) have been applied. More than 52,000 workers have been involved with spill training and cleanup efforts [National Institute for Occupational Safety and Health (NIOSH) 2010a], and BP estimates that the cleanup is about 90% complete (Heron R, personal communication). However, others question this estimate [Georgia Sea Grant 2010; National Oceanic and Atmospheric Administration (NOAA) 2010]. In addition, there are concerns about effects of oil on oxygen levels in the water and the ability of bacteria and other organisms to break down oil and dispersants at all water depths.

Data are available on acute health effects in humans exposed to 7 of 39 large oil spills documented throughout the world (reviewed by Aguilera et al. 2010). These relatively short-term cross-sectional studies have documented acute disorders, including low back pain, headache, inflammation of the eyes and throat, nausea, injuries, lower respiratory tract effects, and psychological effects including depression. To date, workers involved in fishing and clean-up in Louisiana—the most heavily impacted state—have reported headaches, nausea, breathing difficulties, cough, throat irritation, eye irritation, and injuries (Lousiana Department of Health and Hospitals 2010) consistent with acute effects documented in past spills (Aguilera et al. 2010). As of 8–14 August 2010, 376 persons in Louisiana had reported health complaints. Nationally, there have been 2,130 acute health complaints related to this spill (NIOSH 2010b), and as of 7 September 2010, U.S. poison centers had taken 1,155 calls concerning exposure to an oil spill–related toxicant, such as oil, dispersant, contaminated food, or associated toxicants (American Association of Poison Control Centers 2010). In addition, two studies have reported that mental stress and physical symptoms have been experienced by 35–45% of Gulf Coast respondents (Abramson et al. 2010; Lee et al. 2010).

Heavy fuel oils are classified as possible human carcinogens [International Agency for Research on Cancer (IARC) 1989], and there is ample evidence in laboratory animal studies that heavy oil is carcinogenic (Marathon Petroleum Company 2005). Further, many individual substances found in crude oil, including benzene, benzo[*a*]pyrene, and arsenic are class 1 human carcinogens (IARC, in press), and other chemicals in the oil and dispersants may also be carcinogenic. In addition, exposure to heavy oil and related contaminants may be able to increase cancer risk indirectly through, for example, mechanisms related to stress (Reiche et al. 2004), immunosuppression (Grulich et al. 2007), or endocrine disruption (Soto and Sonnenschein 2010).

Although long-term follow-up is essential to assess potential impacts of oil spill exposures on cancer and other chronic diseases, essentially no long-term epidemiologic studies have been reported in the literature, nor has population research been conducted on the health effects of exposure to oil, its breakdown products, and dispersants via the food chain.

Long-term study is also needed to examine effects of these exposures on pregnant women and their infants. Bis(2-ethylhexyl) phthalate, which is present in both Corexit 9500 and Corexit 9527, has been associated with testicular effects, fertility, toxicity to kidneys, and cancer (Agency for Toxic Substances and Disease Registry 2002; Tickner et al. 2001). Benzene exposure during pregnancy has been associated with low birth weight and childhood leukemia, and exposures to polyaromatic hydrocarbon have been associated with low birth weight and small head circumference (Slama et al. 2009; U.S. Environmental Protection Agency 2009); other outcomes of potential concern include spontaneous abortion, birth defects, and aneuploidy (Eskenazi B, personal communication).

There are many challenges to designing and performing optimal epidemiologic studies in this environment: Meaningful community involvement is essential so concerns can be addressed, and accurate documentation is needed for individuals involved in spill cleanup or who live in the impacted areas (e.g., a register). This cohort should include oil rig and response workers (both exposed and presumably unexposed workers); cleanup workers on ships and on the shore; first responders, including health care workers; and representative individuals and families from the general population, with special attention to those involved in maritime/marine activities.

A cohort study requires a long-term commitment from both study participants and researchers, and it must represent rigorous science and be conducted in a manner that protects the rights of individual participants. A study of this cohort should include quantitative exposure estimation through biomonitoring, genotoxicity studies, risk assessments, and other techniques, and determination of intermediate and long-term effects; it should also assess the potential for ongoing exposures via contamination of the food chain and the disposal of oil/dispersant–soaked material in landfills or through burning. Exposure is time dependent; thus, identification and enrollment of the workers, their families, and the communities need to begin now with the collection of basic epidemiologic, occupational, and baseline health data, as well as collection and banking of biological samples from participants. A multidisciplinary team of scientists, including epidemiologists, biostatisticians, biochemists, and other environmental scientists, is needed to contribute to the design, conduct, and interpretation of these human health studies.

It is both reasonable and responsible to be concerned about cancer and other diseases of long latency arising from this unfortunate incident, and we have the tools and expertise to evaluate these health concerns throughout the impacted states. All parties must act with expedience, professionalism, and commitment to the populations of the Gulf South.

## Figures and Tables

**Figure:**
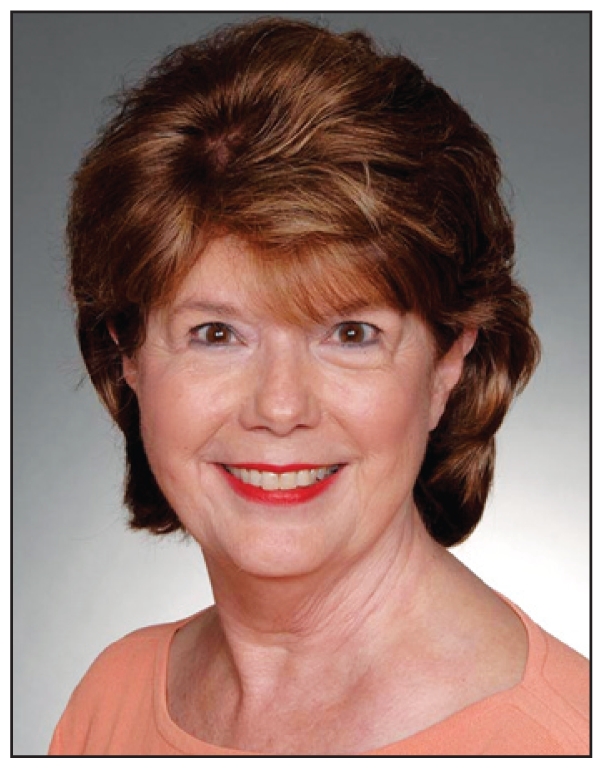
Elizabeth T.H. Fontham

**Figure:**
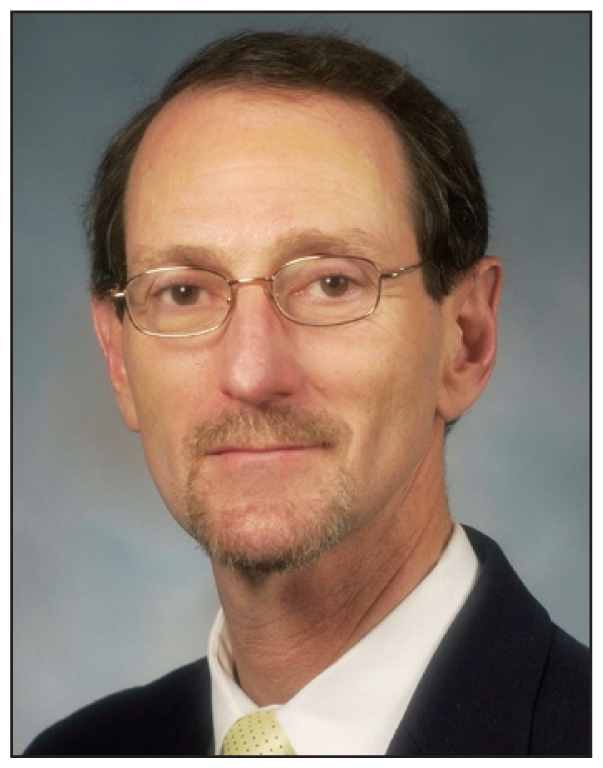
Edward Trapido
