# Errata

**Published:** 2010-11

In the editorial by Fontham and Trapido [ Environ Health Perspect 118:A422–A423 (2010)], one reference cited in the text was inadvertently omitted from the reference list. The reference for Grulich et al. (2007) is as follows:

Grulich AE, van Leeuwen MT, Falster MO, Vajdic CM. 2007. Incidence of cancers in people with HIV/AIDS compared with immunosuppressed transplant recipients: a meta-analysis. Lancet 370(9581):59–67.

*EHP* apologizes for the error.

